# A Dual Role for Macrophages in Modulating Lung Tissue Damage/Repair during L2 *Toxocara canis* Infection

**DOI:** 10.3390/pathogens8040280

**Published:** 2019-12-02

**Authors:** Berenice Faz-López, Héctor Mayoral-Reyes, Rogelio Hernández-Pando, Pablo Martínez-Labat, Derek M. McKay, Itzel Medina-Andrade, Jonadab E. Olguín, Luis I. Terrazas

**Affiliations:** 1 Unidad de Biomedicina, Facultad de Estudios Superiores Iztacala, Universidad Nacional Autónoma de México (UNAM), Tlalnepantla 54090, MEX, México; berefaz@comunidad.unam.mx; 2 Servicio de Medicina Interna, Hospital General de México Dr. Eduardo Liceaga, Ciudad de México 06720, México; hectormayoral@comunidad.unam.mx; 3 Departamento de Patología, Sección de Patología Experimental, Instituto Nacional de Ciencias Médicas y Nutrición, Salvador Zubirán, Ciudad de México 14000, México; rogelio.hernandezp@incmnsz.mx; 4 Laboratorio de Parasitología, Facultad de Estudios Superiores Cuautitlán, UNAM, Cuautitlán I 54716, MEX, México; jpmlabat@servidor.unam.mx; 5 Gastrointestinal Research Group and Inflammation Research Network, Department of Physiology and Pharmacology, Calvin, Joan and Phoebe Snyder Institute for Chronic Diseases, Cumming School of Medicine, University of Calgary, Calgary, AB T2N 4N1, Canada; dmckay@ucalgary.ca; 6 Laboratorio Nacional en Salud, Facultad de Estudios Superiores Iztacala, UNAM, Tlalnepantla 54090, MEX, México; xoxo_pixar@comunidad.unam.mx (I.M.-A.); e.olguin@comunidad.unam.mx (J.E.O.)

**Keywords:** STAT1, STAT6, M1-M2 macrophages, tissue damage-repair, parasite resistance

## Abstract

Macrophages that are classically activated (M1) through the IFN-γ/STAT1 signaling pathway have a major role in mediating inflammation during microbial and parasitic infections. In some cases, unregulated inflammation induces tissue damage. In helminth infections, alternatively activated macrophages (M2), whose activation occurs mainly via the IL-4/STAT6 pathway, have a major role in mediating protection against excessive inflammation, and has been associated with both tissue repair and parasite clearance. During the lung migratory stage of *Toxocara canis*, the roles of M1 and M2 macrophages in tissue repair remain unknown. To assess this, we orally infected wild-type (WT) and STAT1 and STAT6-deficient mice (STAT1^−/−^ and STAT6^−/−^) with L2 *T. canis*, and evaluated the role of M1 or M2 macrophages in lung pathology. The absence of STAT1 favored an M2 activation pattern with Arg1, FIZZ1, and Ym1 expression, which resulted in parasite resistance and lung tissue repair. In contrast, the absence of STAT6 induced M1 activation and iNOS expression, which helped control parasitic infection but generated increased inflammation and lung pathology. Next, macrophages were depleted by intratracheally inoculating mice with clodronate-loaded liposomes. We found a significant reduction in alveolar macrophages that was associated with higher lung pathology in both WT and STAT1^−/−^ mice; in contrast, STAT6^−/−^ mice receiving clodronate-liposomes displayed less tissue damage, indicating critical roles of both macrophage phenotypes in lung pathology and tissue repair. Therefore, a proper balance between inflammatory and anti-inflammatory responses during *T. canis* infection is necessary to limit lung pathology and favor lung healing.

## 1. Introduction

Toxocariasis is a neglected disease despite being broadly distributed, and there has been limited attention given to prevention, treatment, and disease surveillance [1]. Adult *Toxocara canis* worms live in the small intestine of dogs, which excrete eggs in the feces, polluting the environment [2]. Once in the small intestine of intermediate hosts (it can be paratenic hosts, such as humans or mice), larvae penetrate the gut wall and migrate to many organs, including the lung, liver, muscles, and central nervous system, resulting in significant pathology. Infection in the lung with *T. canis* L2 larvae can cause an asthma-type condition in children [3,4]; airway hyperresponsiveness, pulmonary inflammation, and increased levels of IgE can persist for months in mice experimentally-infected with *T. canis* [5,6]. Thus, assessing the inflammatory response, including defining roles for cells such as classically activated macrophages (M1) in the pathology following infection with *T. canis* is critical to a better understanding of the disease and the development of targeted therapies.

M1 macrophages are primarily activated via interferon (IFN)-γ through signaling pathways, such as signal transducer and activator of transcription 1 (STAT1), and interferon regulatory factors (IRF) 1 and 8, which allow expansion of the inflammatory immune response [7,8]. M1 macrophages produce pro-inflammatory cytokines such as TNF-α, IL-1β, IL-6, IL-12, and IL-23 and nitric oxide (NO) through the enzyme inducible nitric oxide synthase (iNOS) [9], and have high expression of the co-stimulatory CD80 and CD86 molecules [10]. On the other hand, during helminth-infection, there are both potent type 2 and immunoregulatory networks that can limit tissue damage caused by the parasite (or the immune response against them). A cellular hallmark of anti-parasitic type 2 immunity is alternatively activated macrophages (M2), activated by interleukin (IL)-4 and IL-13 through the IL-4 receptor alpha (IL-4Rα), and mobilization of STAT6, c-Myc, and IRF4 [7,11,12,13]. Thus, M2 activation promotes control of helminth infection and tissue repair [14,15]. STAT6 regulates many of the genes associated with mouse M2 macrophages, including arginase 1 (Arg1), resistin-like-α (Retnlα, Relmα or FIZZ1), chitinase 3-like 3 (Chi3L3 or Ym1), and macrophage mannose receptor (MMR) or CD206. M2 macrophages have the capacity to block iNOS, counteracting tissue damage caused by M1 macrophages [8,10]. Furthermore, M2 macrophages activated via STAT6 are an important source of chemokines, cytokines, elastin, matrix metalloproteinases (MMPs), and other mediators that drive cellular responses following tissue injury [16,17].

Several studies with nematode-infected mice demonstrate the relevance of the balance between M1 and M2 macrophages and the molecules these cells release [18]. Notably, studies involving *Heligmosomoides polygyrus* [19] and *Nippostrongylus brasiliensis* [20], as well as the filarial nematodes *Brugia malayi* and *Litomosoides sigmodontis* [21], and the trematode *Schistosoma japonicum* [22] highlight an initial T-helper 1 (Th1)-M1 inflammation profile associated with tissue damage, followed by a Th2-M2 polarization that promotes tissue repair. It is clear that macrophages participate in many important immune functions following infection with helminths that are characterized by M2 signature molecules (e.g. including Arg1, FIZZ1, and Ym1) [23]. However, there are limited data regarding the implication of macrophage activation via STAT1 and STAT6 during the second larvae (L2) infective stage of *T. canis* infection. Previously, we reported that Th2 cytokines have a dual role during toxocariasis. On the one hand, STAT6 activation contributes to host susceptibility, and, alternatively, the down-regulated immune response reduces the immunopathology induced by *T. canis infection* in the lungs [24].

Despite these findings, many unresolved questions remain. It has not been determined whether M2 macrophages are the major cell-type involved in tissue repair through their activation via STAT6. Also, the role of M1 macrophages after L2 *T. canis* infection remains unknown. To better understand the possible roles played by M1 and M2 macrophages during acute toxocariasis, we used STAT1- or STAT6-deficient mice. Following L2 *T. canis* infection, STAT1^−/−^ mice favored M2 activation defined by increased lung expression of Arg1, FIZZ1, and Ym1, which resulted in parasite resistance and lung tissue repair. The STAT6^−/−^ mice displayed evidence of M1 activation and iNOS expression, which helped reduce the number of larvae in different tissues, but generated increased inflammation and extensive lung pathology. Therefore, M1 and M2 macrophages are involved in *T. canis*-associated pathology. We suggest that during the lung migratory stage of *T. canis* a balance at a specific time between the inflammatory and anti-inflammatory response is necessary for protection against *T. canis*.

## 2. Results

### 2.1. Parasite Burden and Kinetics of Parasite Migration

**Figure 1:**
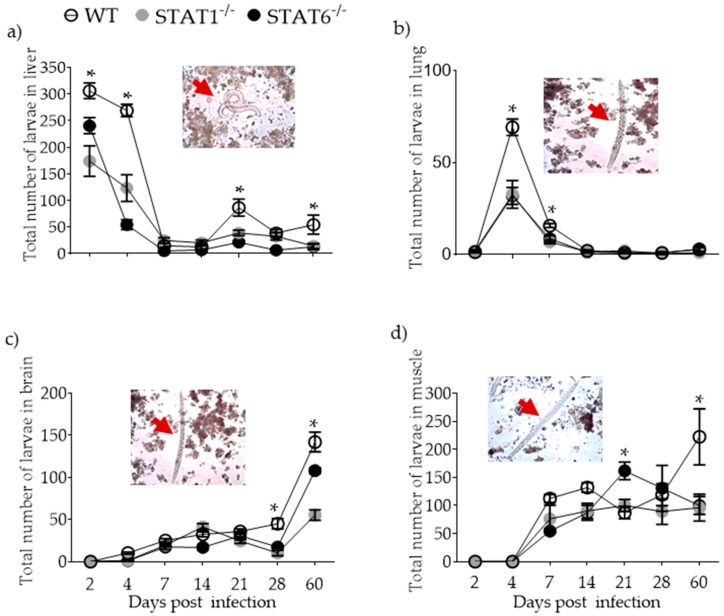
Kinetics of parasite migration. After infection with 500 L2 *T. canis* nematodes, the parasites were counted in the liver (**a**) lungs (**b**) brain (**c**) and muscle (**d**) in wild-type (WT) (solid white circles), STAT1^−/−^ (STAT1 deficient mice, solid gray circles) and STAT6^−/−^ (STAT6 deficient mice, solid black circles) mice at 2, 4, 7, 14, 21, 28, and 60 dpi. Red arrows indicate the presence of larvae in each tissue during the counting. Data are shown from two independent experiments as the mean ± SEM (n = 6 per group); two-way ANOVA with Tukey multicomparison test; * *p* < 0.05 comparing WT versus STAT1^−/−^ and STAT6^−/−^, and STAT1^−/−^ versus STAT6^−/−^ mice, at the same time point of infection.

To assess susceptibility (presence) or resistance (absence) to *T. canis* larvae, BALB/c WT, STAT1^−/−^ and STAT6^−/−^ mice were orally infected with 500 L2 larvae and the kinetics of parasite burden in the liver, lung, brain, and muscle was determined. Wild-type mice showed highest parasite burden in the liver and lung early in infection (4 dpi; Figure 1a,b) and diminishing thereafter. The brain and muscle were larger devoid of parasites at 4 dpi and increased gradually in these tissues over the next 56 days (Figure 1c,d). In contrast, although STAT1^−/−^ and STAT6^−/−^ mice displayed the same larval migration patterns as that of WT mice, the parasite burden in both strains was lower compared to WT mice. These data suggest that the absence of STAT1 and STAT6 did not affect the migration pattern of the larvae and that both STATs are crucial in eliminating the tissue stages of *T. canis*, making both STAT1^−/−^ and STAT6^−/−^ mice less susceptible to L2 *T. canis* infection.

### 2.2. Hemorrhagic Lesions and Inflammatory Infiltrates in Lung Tissue are Affected by STAT1 and STAT6

**Figure 2:**
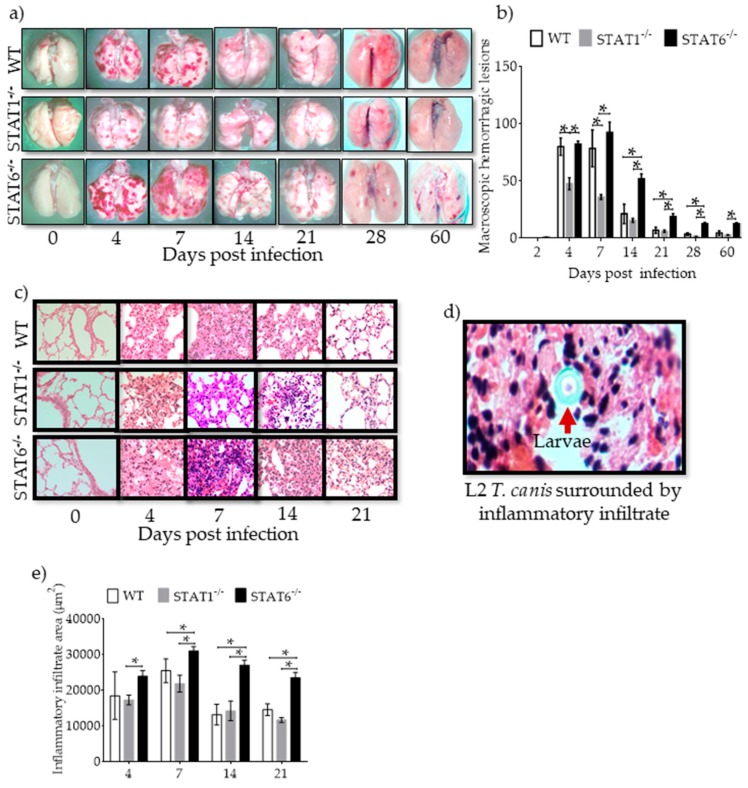
Hemorrhagic lesions and inflammatory infiltrates in lung tissue. Lungs were collected, washed with PBS and photographed at 0–60 dpi (**a**) and macroscopic hemorrhagic lesions were counted in WT (white bars), STAT1^−/−^ (gray bars) and STAT6^−/−^ (black bars) mice (**b**). Paraffin sections 4 µm thick from lung tissue were H&E stained, and inflammatory infiltration and hemorrhagic lesions were observed in microscopy light (40× objective) images at 0, 4, 7, 14, and 21 dpi in the three strains of mice (**c**). Representative image of the L2 *T. canis* surrounded by inflammatory infiltrate (60× objective) (**d**). Inflammatory infiltration was quantified from images taken at 40× objective in light microscope (**e**) in WT (white bars), STAT1^−/−^ (gray bars) and STAT6-/- (black bars) mice. Data are shown from two independent experiments as the mean ± SEM (n = 6 per group). Two-way ANOVA with Tukey’s multicomparison test. * *p* < 0.05 comparing WT versus STAT1^−/−^ and STAT6^−/−^, and STAT1^−/−^ versus STAT6^−/−^ mice at the same time point of infection.

Having confirmed the migration of L2 *T. canis* through the lungs, lung pathology was assessed during acute infection. We observed the development of numerous hemorrhagic spots in the lung parenchyma of WT mice on 4 and 7 dpi, which gradually decreased over time (Figure 2a,b). Following infection with L2 *T. canis*, STAT1^−/−^ mice showed fewer and smaller lesions in the lungs on 4 and 7 dpi as compared to WT mice: lung lesions were not detectable on 28 dpi in STAT1^−/−^ mice (Figure 2a,b). However, STAT6^−/−^ mice, despite having fewer larvae than WT mice (and similar to parasite burden of STAT1^−/−^ mice), displayed numerous hemorrhagic lesions in the lungs that persisted until 60 dpi (end of experiment) (Figure 2a,b). Histological examination of the lungs revealed peribronchial and perivascular inflammation during acute infection (4–7 dpi) that healed as the infection progressed in both WT and STAT1^−/−^ mice. Erythrocytes in the alveolar space and the presence of larvae surrounded by inflammatory infiltrate (Figure 2c–e) correlated with the hemorrhagic lesions observed in the macroscopic study. Interestingly, histological analysis of STAT6^−/−^ mouse lungs revealed a marked inflammatory infiltrate that lasted for longer periods of time (4 to 60 dpi), suggesting impaired healing, despite parasite clearance. These findings suggest that the prompt or delayed healing of the lungs in STAT1^−/−^ or STAT-6^−/−^ mice, respectively, was associated with the type of immune response elicited, rather than the presence of L2 *T. canis* parasitic burden. Enumeration of lymphocytes (Figure S3a), macrophages (Figure S3b) and polymorphonuclear (PMN) (Figure S3c) cells revealed greater numbers of macrophages in lung tissue from day 4 to 21 post infection in WT, STAT1^−/−^ and STAT6^−/−^ mice.

### 2.3. Immune Response Associated with Lung Tissue Damage

**Figure 3:**
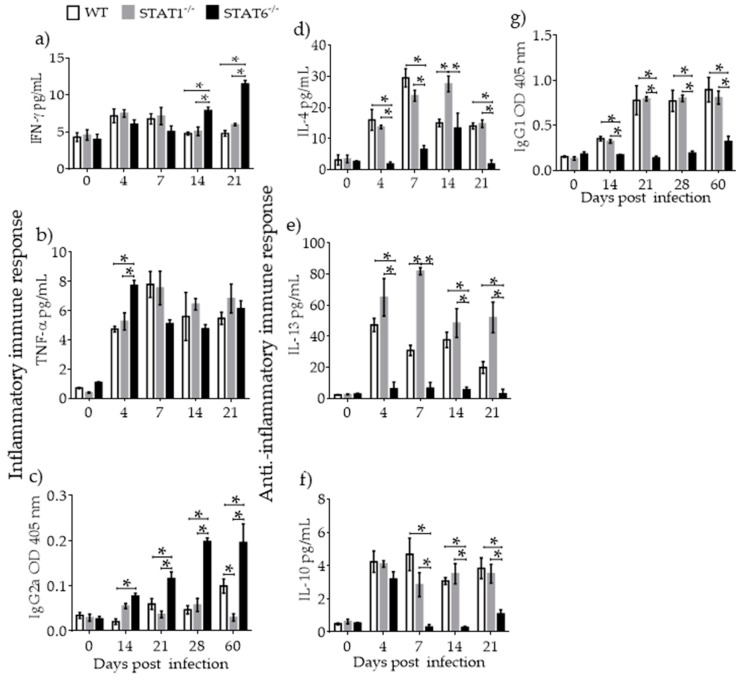
Immune response associated with lung tissue damage during *T. canis* infection. Cytokines and antibodies of inflammatory (interferon (IFN)-γ (**a**), TNF-α (**b**), IgG2a (**c**)) and anti-inflammatory IL-4 (**d**), IL-13 (**e**), IL-10 (**f**), IgG1 (**g**) immune responses were measured in serum at 0–60 dpi from WT (white bars), STAT1^−/−^ (gray bars) and STAT6^−/−^ (black bars) mice. Data are shown from two independent experiments as the mean ± SEM. For cytokines: (n = 4–6 per group); unpaired t-test with Holm–Sidak multicomparison test. For antibodies: (n = 6 per group); two-way ANOVA with Tukey’s multicomparison test. * *p* < 0.05 comparing WT versus STAT1^−/−^ and STAT6^−/−^, and STAT1^−/−^ versus STAT6^−/−^ mice at the same time point of infection.

To determine whether an inflammatory or anti-inflammatory immune response profile accompanied the delayed recovery of lung tissue in *T. canis*-infected STAT6^−/−^ mice, cytokines and IgG isotypes of anti-*T. canis*-specific antibodies were measured in peripheral blood serum. Inflammatory cytokines such as IFN-γ (Figure 3a) and TNF-α (Figure 3b), as well IgG2a (Figure 3c), were increased in *T. canis*-infected STAT6^−/−^ mice compared to WT and STAT1^−/−^ mice (Figure 3a–c). The type 2 cytokines, IL-4 (Figure 3d), IL-10 (Figure 3e), and IL-13 (Figure 3f) and IgG1 (Figure 3g), were increased in WT and STAT1^−/−^ mice but not STAT6^−/−^ mice.

**Figure 4:**
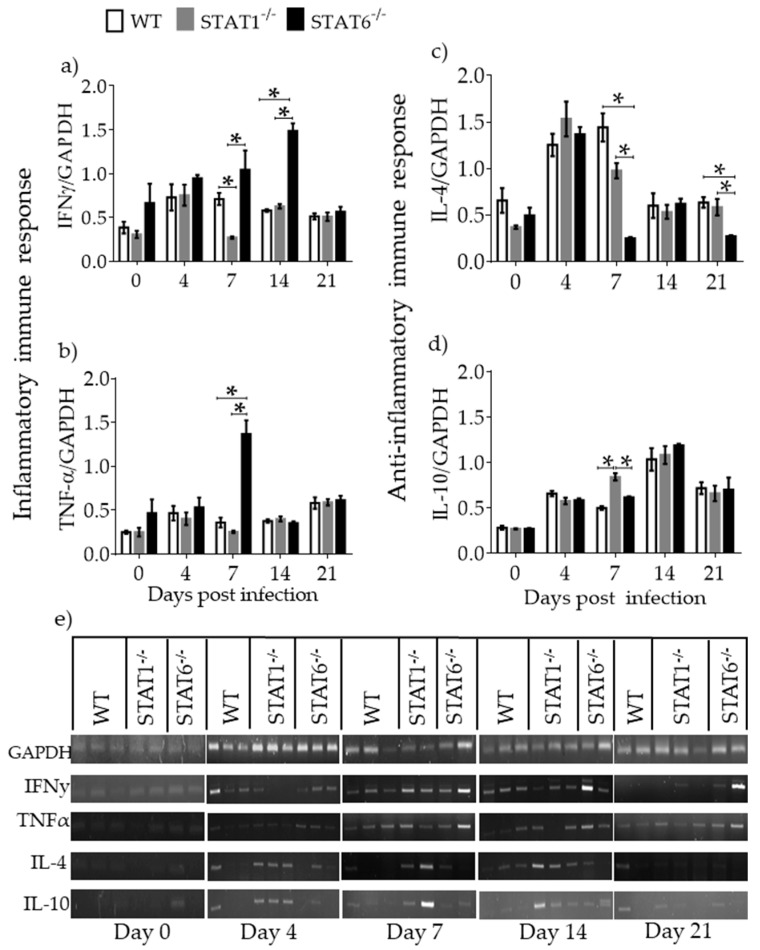
Local immune response associated with lung tissue damage. mRNA expression of IFN-γ (**a**), TNF-α (**b**), IL-4 (**c**) and IL-10 (**d**) was evaluated in whole lung tissue of WT (white bars), STAT1^−/−^ (gray bars) and STAT6^−/−^ (black bars) mice at 0, 4, 7, 14, and 21 dpi. A representative electrophoresis gel of the various genes and GAPDH control from one representative experiment is shown (**e**) Data are shown from two independent experiments as the mean ± SEM (n = 5–6 per group); unpaired t-test with Holm–Sidak multicomparison test.; * *p* < 0.05 comparing WT versus STAT1^−/−^ and STAT6^−/−^, and STAT1^−/−^ versus STAT6^−/−^ mice at the same time point of infection.

To assess the local immune response, cytokines were measured by RT-PCR in whole lung tissue. *T. canis*-infected STAT6^−/−^ mice had increased expression of IFN-γ (Figure 4a) and TNF-α (Figure 4b) at the time when major macroscopic lesions occurred in the lungs. On the other hand, STAT1^−/−^ mice displayed increased expression of IL-4 (Figure 4c) and IL-10 (Figure 4d,e), matching the reduced number of macroscopic lesions in the lungs. These results support the hypothesis that the delayed recovery in lung tissue from STAT6^−/−^ mice could be associated with a pro-inflammatory profile, while a prompt recovery in STAT1^−/−^ mice may be associated with an anti-inflammatory profile during L2 infection with *T. canis*.

### 2.4. M1 and M2 Signature Activation Markers in Lung Tissue during Acute T. canis Infection

**Figure 5:**
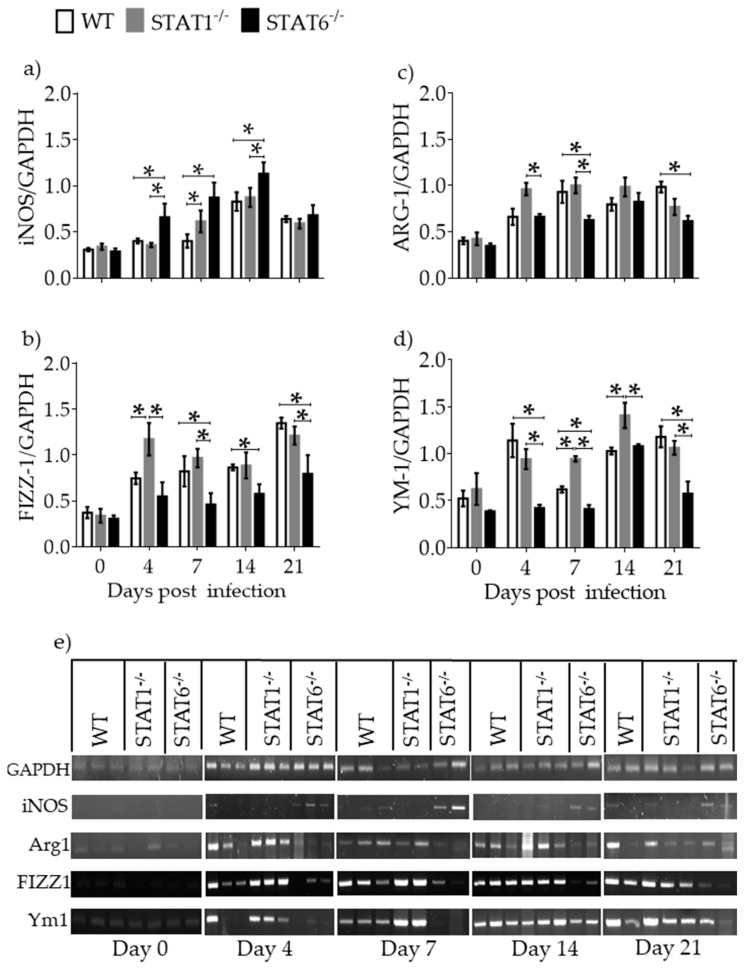
Classically activated (M1) and alternatively activated (M2) macrophage activation markers in lung tissue. mRNA expression of inducible nitric oxide synthase (iNOS) (**a**) Arg1 (**b**) FIZZ1 (**c**) and Ym1 (**d**) was evaluated by RT-PCR in whole lung tissue from WT (white bars), STAT1^−/−^ (gray bars) and STAT6^−/−^ (black bars) mice at day 0, 4, 7, 14, and 21 dpi. A representative electrophoresis gel of the different previously mentioned genes and their respective GAPDH control from one experiment is shown (**e**) Data from graphs are shown from two independent experiments as the mean ± SEM (n = 5–6 per group); unpaired t-test with Holm–Sidak multicomparison test.; * *p* < 0.05 comparing WT versus STAT1^−/−^ and STAT6^−/−^, and STAT1^−/−^, versus STAT6-/- mice at the same time point of infection.

IFN-γ is critical for M1 macrophage activation and iNOS expression [7,9]. IL-4 and IL-13 are important for M2 macrophage differentiation and Arg1, FIZZ1, and Ym1 expression [17]. Thus, mRNA for these M1 and M2 markers were measured in lung samples by RT-PCR. iNOS mRNA expression was markedly increased in *T. canis*-infected STAT6^−/−^ mice (Figure 5a) corresponding temporally with the appearance of macroscopic lesions in the lungs. In contrast, the mRNAs for Arg1, FIZZ1 and Ym1 were increased in the lungs of WT and STAT1^−/−^ mice (Figure 5b–e).

**Figure 6:**
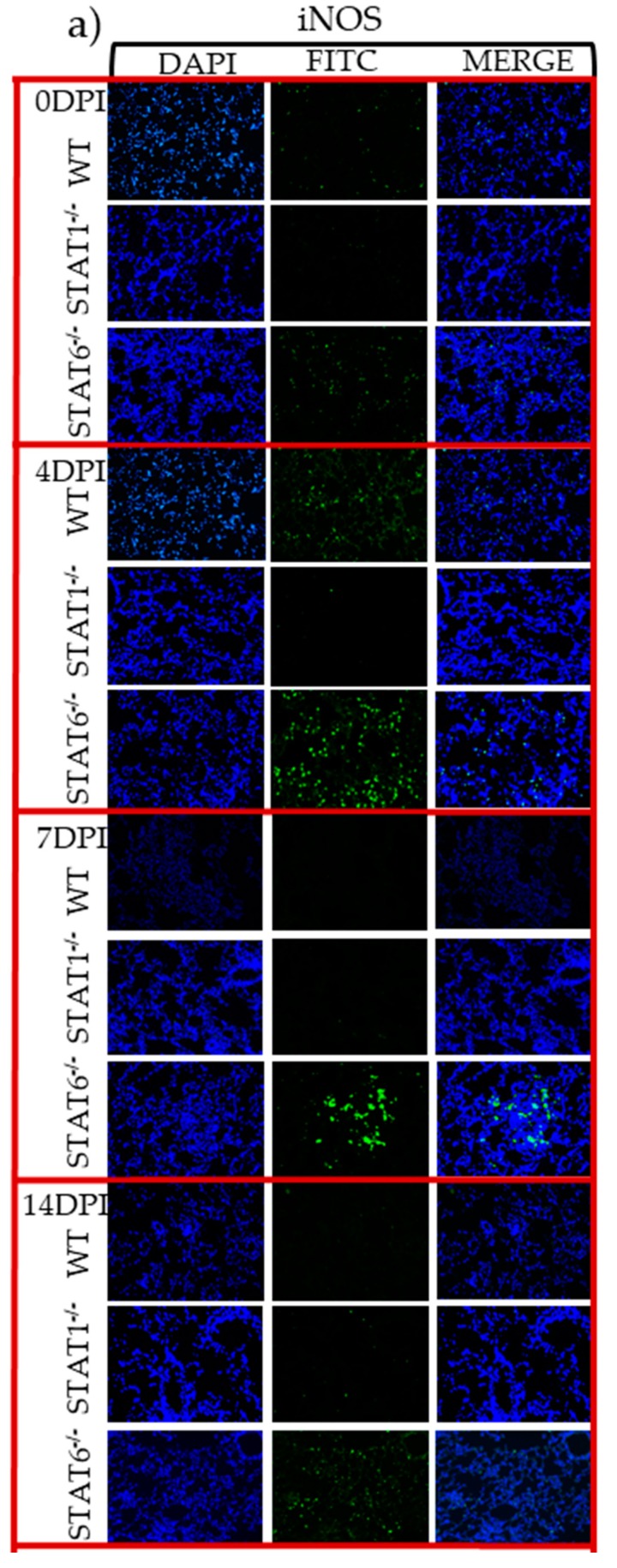

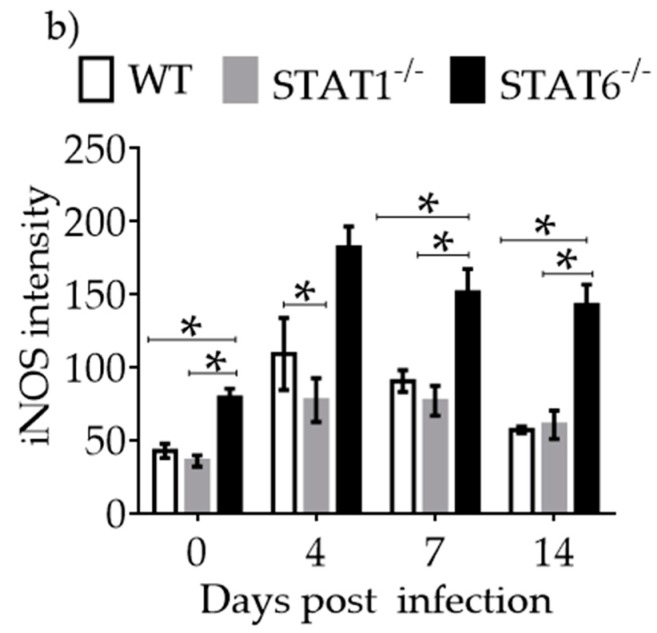
M1 macrophage activation marker in lung tissue assessed by immunofluorescence. Microscopy data of lung sections from WT, STAT1^−/−^ and STAT6^−/−^ mice at 4, 7 and 14 dpi, stained with the DNA-binding dye (DAPI) in blue and iNOS (**a**) in green. Quantification of the fluorescent intensity of iNOS (**b**) in lung sections stained from a, in WT (white bars), STAT1^−/−^ (gray bars) and STAT6^−/−^ (black bars) mice. Photographs were taken with a 20× objective. Data are shown from two independent experiments as the mean ± SEM (n = 6 per group). Two-way ANOVA with Tukey’s multicomparison test. * *p* < 0.05 comparing WT versus STAT1^−/−^ and STAT6^−/−^, and STAT1^−/−^ versus STAT6^−/−^ mice at the same time point of infection.

Lung sections were subsequently assessed by immunofluorescence. iNOS protein expression was strongly detected by 4 dpi, and remained elevated in the lungs it was sustained at least until 14 dpi in STAT6^−/−^ mice (Figure 6a,b). In contrast, STAT1^−/−^ mice had reduced expression of iNOS, while WT mice displayed discrete and transient iNOS expression, particularly at 4 dpi (Figure 6a,b).

**Figure 7:**
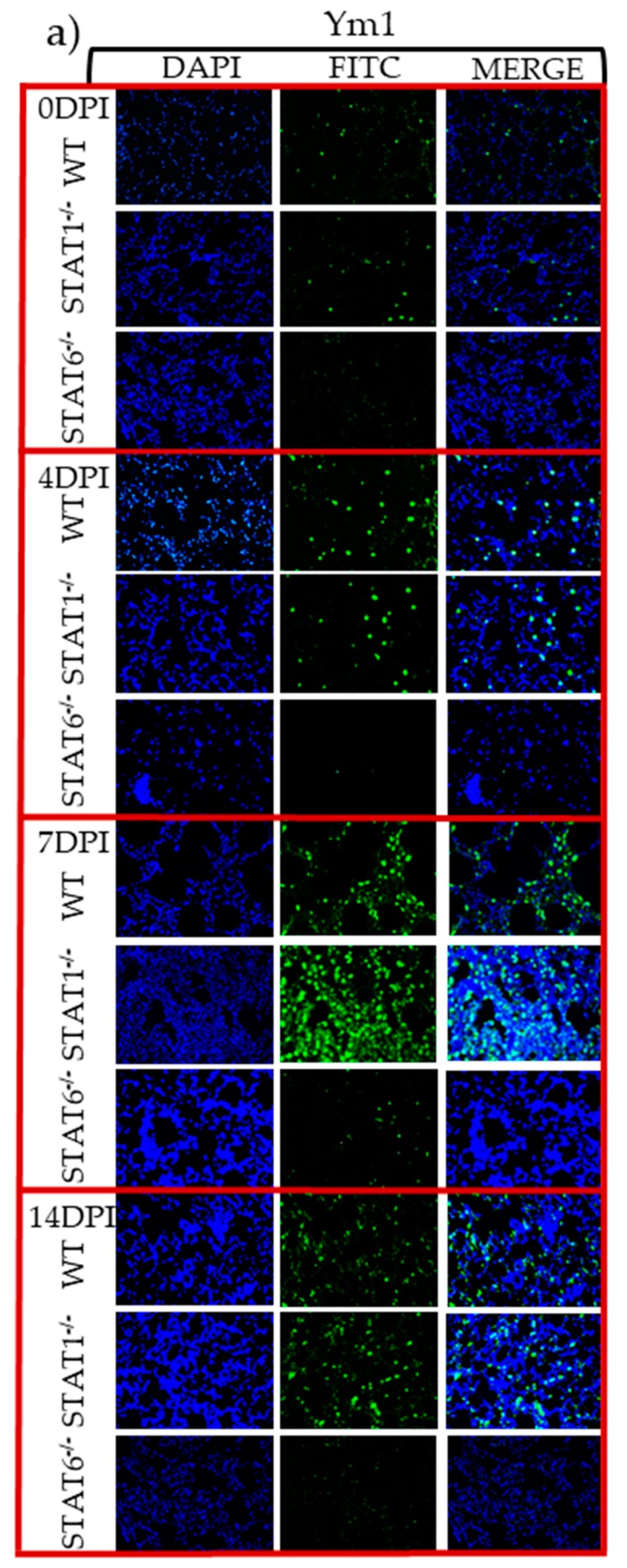

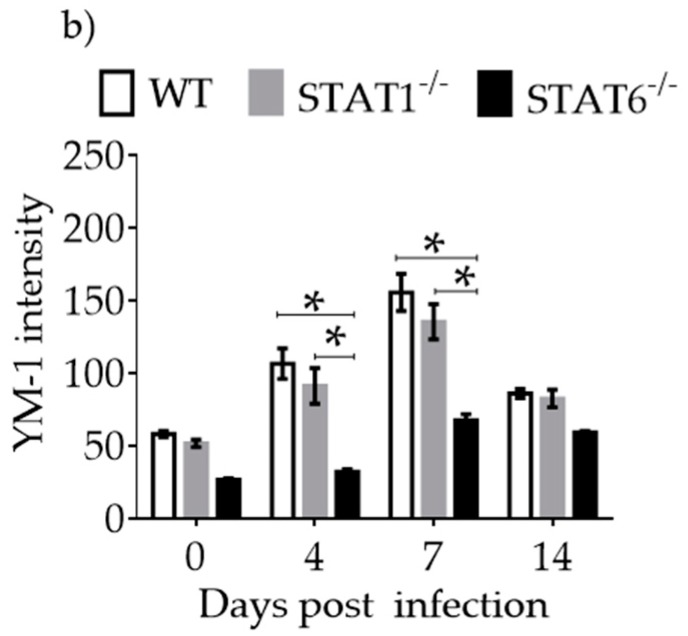
M2 macrophage activation marker in lung tissue assessed by immunofluorescence. Microscopy data of lung sections from WT, STAT1^−/−^, and STAT6^−/−^ mice at 4, 7, and 14 dpi, stained with the DNA-binding dye (DAPI) in blue and Ym1 (**a**) in green. Quantification of the fluorescent intensity of Ym1 (**b**) in lung sections stained from a, in WT (white bars), STAT1^−/−^ (gray bars) and STAT6^−/−^ (black bars) mice. Photographs were taken with a 20× objective. Data are shown from two independent experiments as the mean ± SEM (n = 6 per group). Two-way ANOVA with Tukey’s multicomparison test. * *p* < 0.05 comparing WT versus STAT1^−/−^ and STAT6^−/−^, and STAT1^−/−^ versus STAT6^−/−^ mice at the same time point of infection.

Regarding Ym1 expression, which has been consistently associated with tissue repair [25], we found that its early expression in the lungs was largely dependent on STAT6 signaling, given that Ym1 expression was significantly reduced in the lungs of STAT6^−/−^ mice. Ym1^+^ cells were abundant in both WT and STAT1^−/−^ mice, especially at 4 and 7 dpi (Figure 7a,b).

**Figure 8:**
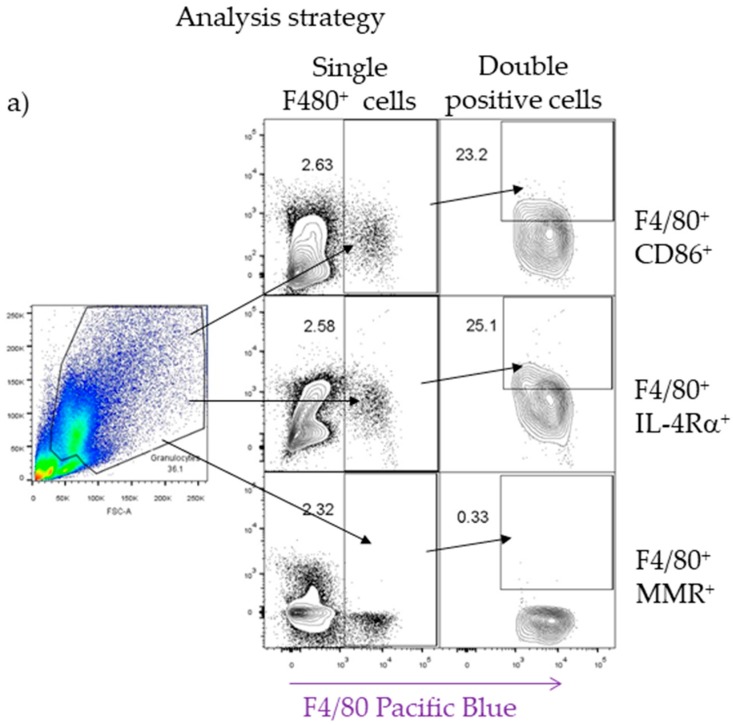

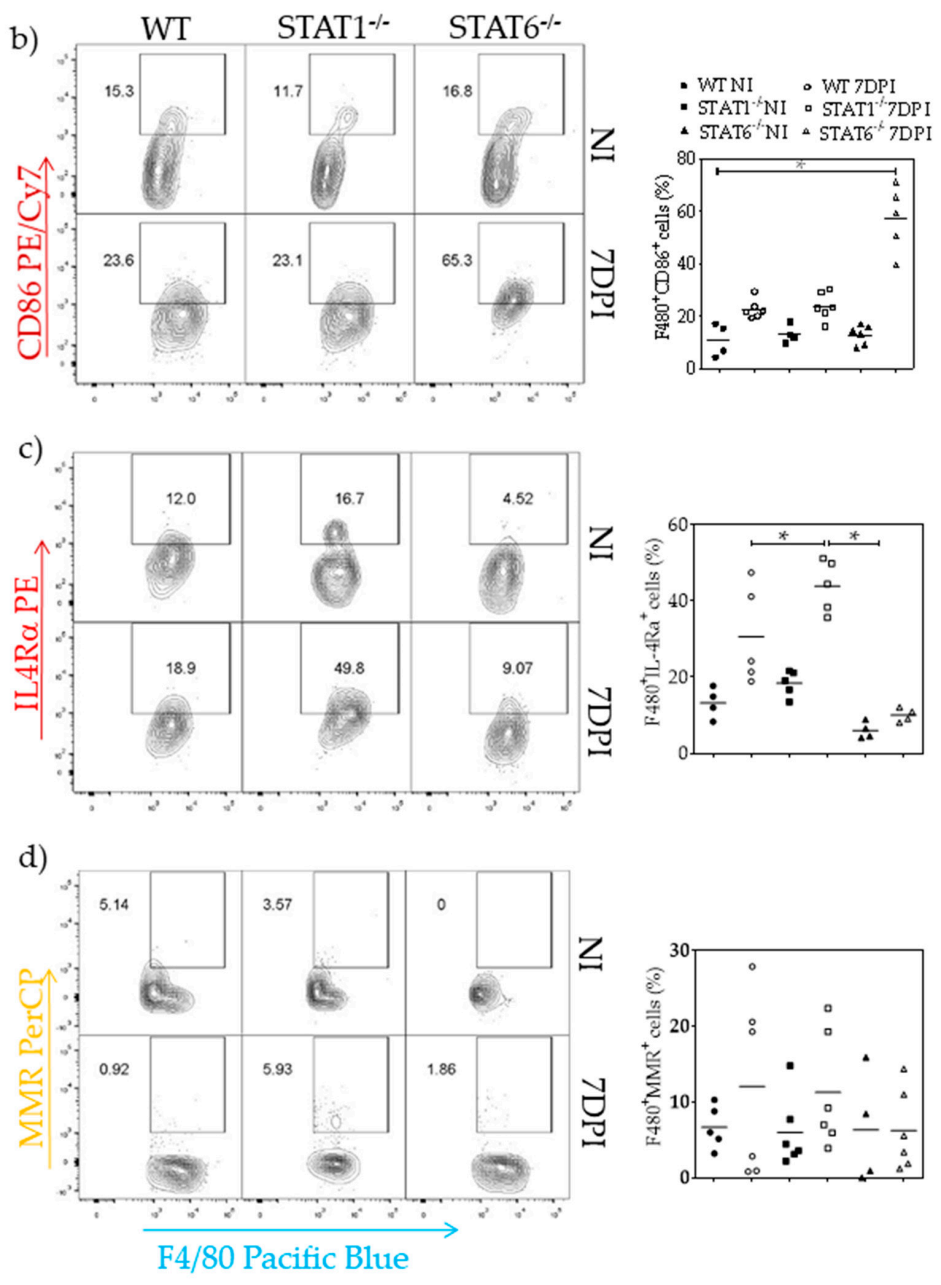
Detection of cell surface markers associated with M1 and M2 macrophage activation. Mice were infected with 500 L2 *T. canis* larvae, and lung cells were obtained at 7 dpi for flow cytometry staining. Representative FACS analysis of the cell surface markers F4/80, CD86, IL-4Rα, and MMR. The macrophage region was first defined by FSC and SSC characteristics and further subgated based on the F4/80 cell population. Then, one thousand events from either subgate were captured (**a**) Representative dot plots and their respective percentage of F4/80^+^CD86^+^ (**b**), F4/80^+^IL-4Rα^+^ (**c**), and F4/80^+^MMR^+^ (**d**) double-positive cells in infected and noninfected (NI) mice. Each dot plot represents an individual mouse. Data are shown from two independent experiments of 5–6 animals per group. One-way ANOVA and Tukey’s multiple comparison test. * *p* < 0.05 comparing WT versus STAT1^−/−^ and STAT6^−/−^ mice.

To confirm the presence of macrophages and to assign them a probable role in either lung tissue damage or repair, F4/80-CD86 double-positive cells for M1 activation and F4/80-IL4Rα and F4/80-MMR cells double-positive for M2 activation were evaluated by flow cytometry (Figure 8a–e). STAT6^−/−^ mice displayed an increased percentage of F4/80^+^CD86^+^ cells that was not observed in either WT or STAT1^−/−^ mice (Figure 8b) at 7 dpi. However, WT and STAT1^−/−^ mice showed an increased percentage of both F4/80^+^IL4Rα^+^ and F4/80^+^MMR^+^ cells (Figure 8c–d) at the same time point after *T. canis* infection.

Collectively, these data suggest that STAT6 deficiency promotes M1 macrophage activation and lung tissue damage, while the absence of STAT1 is associated with M2 macrophage polarization and early lung tissue repair during acute toxocariasis.

### 2.5. Differential Expression of Molecules Associated with Tissue Repair and Fibrosis

**Figure 9:**
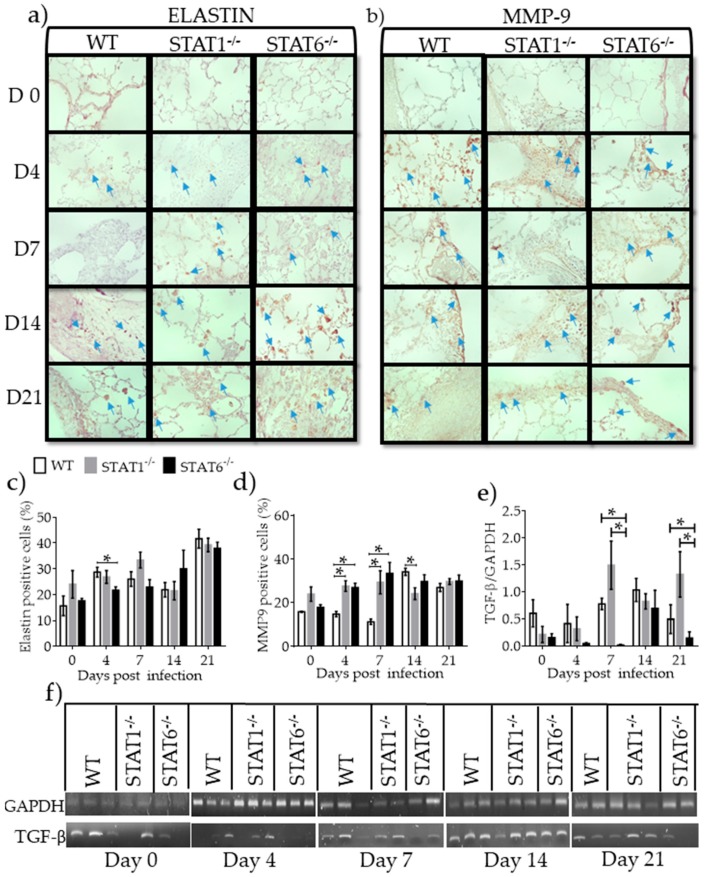
Molecules associated with tissue repair and fibrosis. Paraffin sections of lung tissue were processed to measure elastin (left panel) and MMP9 (right panel) by IC as markers of fibrosis. Representative images from lung tissue sections were photographed with the 60× objective (**a**,**b**) and the percentage of Elastin- and MMP9-positive cells (brown marks pointed by arrows) was measured at different dpi in WT (white bars), STAT1^−/−^ (gray bars) and STAT6^−/−^ (black bars) mice (**c**,**d**) mRNA expression of TGF-β as a marker of tissue repair was measured by RT-PCR (**e**) and its respective electrophoresis gel is shown (**f**) Data are from two independent experiments presented as the mean ± SEM (n = 5–6 per group); unpaired t-test with Holm–Sidak multicomparison test; * *p* < 0.05 comparing WT versus STAT1^−/−^ and STAT6^−/−^, and STAT1^−/−^ versus STAT6^−/−^ mice at the same time point of infection.

Asking if the repair mechanisms contributed to fibrosis generation, factors associated with fibrosis development and tissue repair, namely collagen, elastin, MMP-9, and TGF-β, were assessed. Elastin, a wound repair factor, increased in all strains of mice at 21 dpi. *T. canis*-infected STAT6^−/−^ mice had a smaller increase in elastin compared with WT and STAT1^−/−^ mice at 4 dpi (Figure 9a,c). Masson’s trichrome staining did not reveal differences in collagen deposition between the strains (Supplementary Figure S1). MMP9, an enzyme that degrades elastin, reached peak of expression at 14 dpi in all strains of mice. However, STAT1^−/−^ and STAT6^−/−^ mice displayed higher levels of MMP9 at 4 and 7 dpi (Figure 9b,d). Whereas TGF-β mRNA a factor associated with tissue repair produced by M2 macrophages [18] was modestly expressed in WT mice, its expression was significantly enhanced in STAT1^−/−^ mice at different dpi. Interestingly, STAT6^−/−^ mice failed to enhance TGF-β expression in lung tissue (Figure 9e,f). These observations suggest that neither experimental group of mice promotes greater fibrosis.

### 2.6. Lung Macrophage Depletion Alters the Inflammatory and Repair Process in Acute Toxocariasis

**Figure 10:**
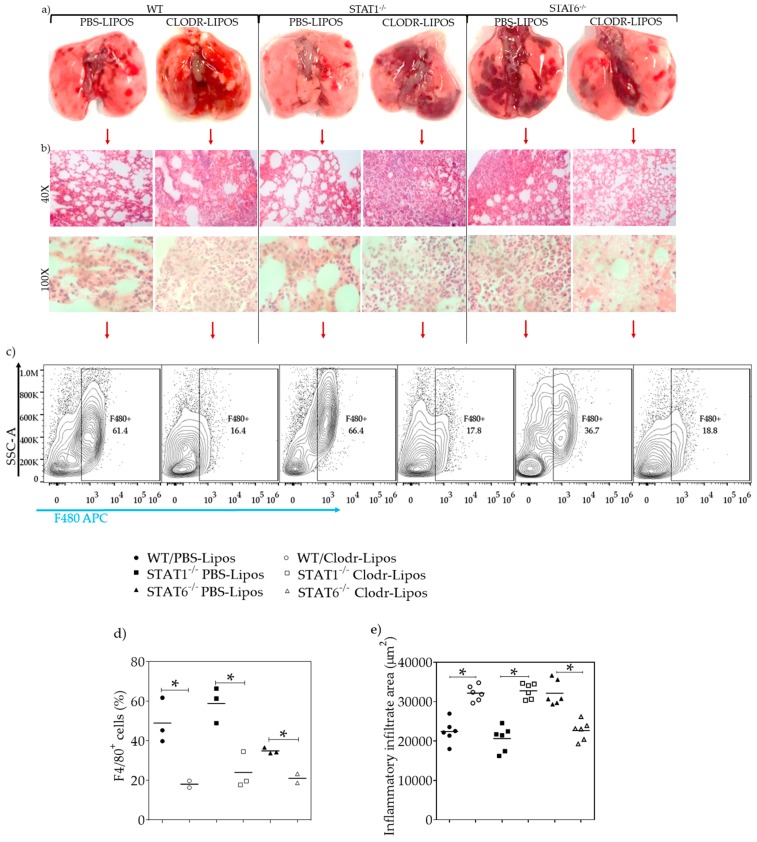
Lung macrophage depletion. WT, STAT1^−/−^, and STAT6^−/−^ mice were treated either with PBS-liposomes or clodronate-liposomes intratracheally to deplete macrophages, before and after being infected with 500 L2 *T. canis* larvae. The animals were euthanized at 4 dpi, and the lungs were taken and photographed, (**a**) H&E staining was carried out to obtain images at 40× or 100× (**b**), and the inflammatory infiltrate areas were measured (**e**). Lungs cells were collected, and flow cytometry was used to determine the efficacy of the treatment. Representative dot plots and their respective percentage of F4/80-positive cells of the three strains of mice (**c**,**d**) * *p* < 0.05 comparing WT versus STAT1^−/−^ and STAT6^−/−^ mice.

To examine whether macrophages were involved in a rapid lung tissue repair process during acute L2 *T. canis* infection, macrophages were depleted by intratracheal administration of clodronate-loaded liposomes. Infected WT and STAT1^−/−^ mice treated with PBS-liposomes displayed well-defined hemorrhagic lesions, while those receiving the clodronate-liposomes displayed severe, large lesions that were more diffuse and widespread throughout the lungs (Figure 10a). In *T. canis*-infected STAT6^−/−^ mice treated with PBS-liposomes, the hemorrhagic lesions were obvious, numerous and widely distributed throughout the lung, compared to WT and STAT1^−/−^ mice (Figure 10a). Conversely, infected STAT6^−/−^ mice treated with clodronate-liposomes developed fewer hemorrhagic lesions compared with those of *T. canis*-infected STAT6^−/−^ and WT mice receiving PBS-loaded liposomes. Notably, administration of clodronate-loaded liposomes in STAT6^−/−^ mice reduced hemorrhagic lesions, and the appearance of the lungs was similar to that of *T. canis*-infected WT and STAT1^−/−^ mice (Figure 10a), suggesting that macrophages may mediate control of hemorrhaging and the inflammation caused by the lung stage of this helminth in a STAT6-dependent manner. In accordance with these findings, inflammatory infiltrates were clearly affected by macrophage depletion. Thus, *T. canis*-infected WT mice treated with clodronate liposomes displayed increased inflammatory infiltrates compared to those of *T. canis*-infected WT mice receiving PBS-liposomes. Similarly, the elimination of macrophages in *T. canis*-infected STAT1^−/−^ mice resulted in an increased inflammatory infiltrate in the lungs. In contrast, *T. canis*-infected STAT6^−/−^ mice treated with clodronate liposomes displayed a marked reduction in inflammatory cell infiltration (Figure 10b,e). Flow cytometry analysis was performed to assess macrophage depletion. Mice receiving clodronate-liposomes had a significant reduction in the F4/80^+^ cell population in all three infected strains of mice, ranging from 50%–74% reduction in lung macrophage numbers compared to that of their infected PBS-liposome control groups (Figure 10c,d). Consequently, analysis of F4/80^+^CD86^+^, F4/80^+^IL4-Rα^+^, and F4/80^+^MMR^+^ double-positive cells revealed no significant differences in either the clodronate liposome-treated groups or between strains (Supplementary, Figure S2).

## 3. Discussion

Macrophage phenotype is dictated by the conditions of the micro-environment and the local cytokine milieu [26]. The M1 macrophage has robust anti-microbial and anti-tumor activity, can cause ROS-induced tissue damage, and impair tissue regeneration and wound healing [27,28]. By contrast, the regulatory anti-inflammatory M2 macrophage promotes tissue restitution after damage [28,29,30,31].

Some authors divided M2 macrophages into wound healing macrophages (M2a) and regulatory macrophages (M2b) [7,32]; others describe macrophage polarization as a “spectrum model” with at least nine distinct macrophage activation programs in humans [33]; Reyes et al [34], Martinez et al, and Mantovani et al [35] reported that M2a macrophages reflects the typical alternative profile induced by IL-4 and IL-13, M2b induced by exposure to immune complexes release high levels of IL-10 and M2c represents the original version of deactivated macrophages induced by IL-10 and glucocorticoid hormones [7,34,35]; Murray et al propose that the term regulatory macrophages should be avoided because all macrophage populations have regulatory capacities and a correct use of the nomenclature concerning M1/M2 is needed to avoid confusions [36]. Following the previous studies and the proper suggestions, for the purpose of the present study we decided to study the common M1/M2 classification, activated by IFN-γ and IL-4 or IL-13, respectively.

Helminth migration through the host on route to its preferred niche can cause significant tissue damage and so it is imperative that the host be capable of initiating rapid and effective tissue repair. Analyses of helminth-rodent model systems has yielded considerable knowledge of the cellular and molecular players responsible for tissue recovery after injury [37,38]. Not surprisingly the mobilization of repair mechanisms is host-parasite specific [39], and several distinct pathways of tissue repair have been described. Yet, there is a paucity of studies of assessing tissue recovery in response to acute toxocariasis [5,6,40].

In the present study, the role of M1 and M2 macrophages in the repair process during the lung stage of *T. canis* infection was assessed using STAT1^−/−^ and STAT6^−/−^ mice. According to the findings, the M1 macrophage population induces damage in the lungs during the early stages of L2 *T. canis* migration. In STAT6^−/−^ mice, even when the larvae were almost absent from the lungs in the chronic stages, tissue damage persisted and correlated with increased iNOS indicative of M1-type cells. Consistent with these observations, macrophage depletion in *T. canis*-infected STAT6^−/−^ mice resulted in a reduction in the number of hemorrhagic lesions in the lungs and better healing. Together, these data strongly suggest that M1 macrophages are a major contributor to lung damage during the early and chronic stages of L2 *T. canis* infection. Despite the damage produced in the absence of STAT6, the mechanisms of tissue repair associated with an anti-inflammatory immune response were not completely suppressed. Thus, markers associated with wound healing such as Arg1, FIZZ1, and Ym1 were detectable, as was TGF- mRNA, and the regenerative process associated with MMP9 and elastin expression were not altered, resulting in delayed, not absent, healing processes in the infected STAT6^−/−^ mice. These data are supported by a recent report from Sutherland et al., showing that Ym1 and FIZZ1 expressions are not completely dependent on the IL4R-α signaling pathway [41].

Other signaling pathways, in addition to STAT6, are implicated in M2 macrophage activation and the repair mechanisms needed when tissues are damaged, that involve, for example, Akt [42], STAT3 [43], IRF, and PPARγ [8] signaling. In additional, epithelial alarmins are secreted following infection with parasitic nematodes to activate type 2 innate lymphoid cells (ILC2) and drive type 2 immunity [37,44,45], which via IL-5 and IL-13 can mobilize wound healing mechanisms with the capacity to produce Arg1 [44] and FIZZ1 [46] to maintain tissue integrity. Consequently, in STAT6^−/−^ mice treated with clodronate-liposomes, depletion of macrophages mainly reduces the population causing inflammation, promoting a more efficient activation of all repair mechanisms and resulting in a reduction in hemorrhagic lesions. Similar results were reported in *N. brasiliensis*-infected STAT6^−/−^ mice where a predominant inflammatory profile was observed coupled to persistence of pathology [47]. Thus, increased inflammation drives incomplete M2 macrophage activation.

In our model of infection with *T. canis*, we evaluated the role of M2 macrophages in lung tissue repair by suppressing the inflammatory response through STAT1^−/−^ mice. We observed that the regenerative process in this strain of mouse was faster compared with STAT6^−/−^ and WT mice. In STAT1^−/−^ mice, an anti-inflammatory profile was apparent; IL-13 expression was increased, Arg1, FIZZ1 and Ym1 were highly expressed (or showed a tendency to be increased), and F4/80^+^IL-4Rα^+^ cells were highly increased and F4/80^+^CD86^+^ cells decreased. Finally, when macrophages were depleted with clodronate-liposomes, major lung pathology manifested. These data from STAT1^−/−^ mice confirm that M2 macrophages play a crucial role in lung tissue healing during *T. canis* infection, and their absence compromises the process of tissue regeneration.

The anti-inflammatory immune response, commonly observed following infection with parasitic helminths, regulates tissue repair via the coordinated activity of Th2 cells, eosinophils, mast cells, basophils, ILCs2, and M2 macrophages [48,49]. Nevertheless, when type 2 cytokine-mediated repair processes become chronic, over exuberant or dysregulated, they can contribute to the development of fibrosis [49]. Diverse mechanisms inducing inflammation and fibrosis resolution are dependent on the etiological agent involved, tissue tropism, and the type of immune response triggered in each case [50]. Other factors associated with tissue repair, such as MMP2 and MMP9, can be produced by M2 macrophages, are involved in keratinocyte migration and contribute to the elimination of nonviable tissue [51,52,53]. In normal conditions, elastin is an essential protein of connective tissue that provides elasticity and support to diverse organs, including the lungs. During pulmonary pathologies, an increase in elastin deposition can cause fibrosis, and to avoid this, MMP9 and MMP2 are activated for protein degradation and restructuring of the extracellular matrix (ECM) [54]. Here, we observed that MMP9 and elastin are produced in similar quantities in all three strains of mice during the early phases of *T. canis*-infection. Thus, it is feasible that such a phenomenon might be a consequence of the damage induced by the helminth, which in turn promotes the activation of mechanisms that are necessary for tissue repair. We speculate that the increase in MMP9 counteracted the effect of elastin and collagen deposition, to avoid an obvious increase in fibrosis. Whereas TGF-β that was mainly expressed in WT and STAT1^−/−^ mice may participate as a regulatory mechanism to facilitate the resolution of pro-inflammatory response and driver of tissue repair [31], therefore, the absence of STAT6 compromise its expression and tissue repair may be by M2 macrophages.

Another novel finding was that the immune response in STAT6^−/−^ mice drove the clearance of the *T. canis* larvae. Previously, we reported corroborating data on parasite burden and concluded that IgE production was not essential for parasite neutralization because STAT6^−/−^ mice did not produce IgE [24]. A possible mechanism underlying the resistance to this parasitic infection in STAT6^−/−^ mice is increased activation of M1 macrophages, which favors iNOS expression. Few studies have proposed a role for NO in the resistance against nematode parasites. In an in vitro study, Pfaff et al. showed that *L. sigmodontis* microfilariae were vulnerable to NO. However, neither pharmaceutical inhibition of nitric oxide synthesis nor iNOS knockout in mice abrogated resistance to circulating *L. sigmodontis* [55]. More recently, iNOS^−/−^ mice infected with *Strongyloides venezuelensis* showed increased susceptibility to infection [56]. These results illustrate that mechanisms of protection or susceptibility defined in one model system cannot be simply assigned to all helminth-host interactions: rather particular differences can be critical in favoring initial inflammatory responses that have a major role in inducing protection during helminth infections. The data herein, can be interpreted in favor of iNOS contributing to the resistance of STAT6^−/−^ mice to infection with L2 *T. canis*.

Moreover, we observed that STAT1^−/−^ mice had a decreased parasite burden, similar to that of STAT6^−/−^ mice. These results can be explained by an exacerbated anti-inflammatory immune response that is the archetypical response against helminth parasites. For instance, FIZZ1 is a reputedly important molecule in eliminating *S. mansoni* or *N. brasiliensis* from the intestine of mice and this effect was linked to an enhanced Th2 immune response [57]. In fact, in the absence of STAT1 we found an acutely increased expression of FIZZ-1, thus it may be that recently hatched larvae are rapidly expelled from the intestine, and therefore lungs, due to an increase in mucus production and fewer larvae may reach blood vessels for dissemination to other organs. In line with this Filbey et al. found that migrating *N. brasiliensis* larvae were killed in the lungs of mice co-infected with *H. polygyrus*, and the lung pathology associated with *N. brasiliensis* larval migration was reduced by robust Th2 immunity [58]. Finally, Esser-von Bieren et al suggested a novel IL-4Rα-independent mechanism of M2 activation that is antibody-dependent and mediates both anti-helminth immunity and tissue disruption caused by migrating *H. polygyrus* larvae [59].

Traditionally eosinophils are cited as an important anti-helminth effector mechanism [60,61,62] but more recent studies suggest the situation is not so simple; while infection with *T. canis* is associated with an eosinophilic response, these cells may have a limited capacity to kill the helminth and the larvae can efficiently escape eosinophils in vitro [63,64,65]. In a recent and interesting review, it has been proposed a combined role for macrophages and eosinophils as key players in the mechanisms of granuloma formation during helminth infections and in the balance between parasite killing and healing [66]. Collectively, a scenario is forming in which inflammatory and anti-inflammatory immune responses are important in mediating tissue and helminth destruction. Thus, during *T. canis* infection, M2 macrophages activated via STAT6 are important cells in modulating lung tissue damage and conferring resistance to infection by inducing a potent anti-inflammatory immune response, whereas M1 macrophages activated via STAT1 promote lung pathology, while at the same time being important for parasite eradication. However, a complementary and more extended study, reviewing the role of other cell populations in conjunction with macrophages that can help to mediate parasite killing and wound healing during L2 *T. canis* infection like eosinophils or ILC2 cells, is needed.

In summary, our work extends awareness of the role(s) of the STAT1 and STAT6 pathways in lung healing during acute *T. canis* infection and highlights the importance of M2 macrophages in accelerating tissue recovery during the lung stage of this infection.

## 4. Materials and Methods

### 4.1. Animals

Eight-to-ten-week-old wild-type (WT), STAT1^−/−^, and STAT-6^−/−^ male BALB/c mice were purchased from the Jackson Laboratory Animal Resources Center (Bar Harbor, ME, USA) and maintained in a specific pathogen-free environment at the FES-Iztacala, U.N.A.M animal facility according to Faculty Animal Care and Use Committee and government guidelines (official Mexican regulation NOM-062-ZOO-1999) in accordance with recommendations in the Guide for the Care and Use of Laboratory Animals of the National Institutes of Health (USA).

### 4.2. Egg Isolation and Parasite Infection

Adult *T. canis* female worms were isolated from the intestines of naturally infected puppies (≤3 months old). Female worms were dissected to isolate immature eggs from the uterus and kept in distilled water. The mixture was washed and centrifuged twice at 1500 rpm for 10 min in 1% NaHCl solution. After removal of the supernatant, the sediment was washed twice with distilled water and placed in a 1% formalin solution according to previous protocols [67] in a tissue flask at 28 °C for one month, with gentle daily agitation until L2 development inside the eggs, which was monitored under the microscope. After maturation, the mice were infected with 500 larvated eggs, administered intragastrically with a Foley tube.

### 4.3. Kinetics of Parasite Migration

Animals were euthanized at 0 (uninfected), 2, 4, 7, 14, 21, 28 and 60 days post infection (dpi). Lungs, liver, muscle and brain were cut into small pieces and placed in tubes containing 1% chloride acid (J.T. Baker; Edo. De Mex, Mexico) and 3% pepsin (Sigma Aldrich; St Louise; USA). To facilitate the release of L2 parasites from the different organs, tissues were incubated for 48 h at room temperature with agitation. After incubation, the samples were centrifuged at 1500 rpm for 10 min, the sediment recovered, reconstituted, and fixed with 10% formalin (Sigma Aldrich; Mexico City, Mexico), and the L2 parasites counted under a 10× objective in an optical microscope (Motic B5 Professional Series).

### 4.4. Macroscopic Lung Study

Complete lungs were taken from the mice, washed with ice-cold saline solution, photographed under a 4x objective using a stereoscopic microscope (ZEIGEN), and hemorrhagic lesions on the surface of the lungs were counted.

### 4.5. Histology, Hematoxylin and Eosin (H&E), and Masson´s Trichrome Staining

The lungs were perfused via trachea and fixed with absolute alcohol, followed by paraffin embedding and 4 μm-thick sections were taken through the hilum and stained with the H&E for conventional histology analysis or Masson´s trichrome stain to observe collagen deposition. Morphological features were assessed using an optical microscopic (LAS V4.9, Leica), and morphometric analysis was performed with the Leica program to determine the total surface and calculate the area of inflammatory infiltrate per µm^2^. Cell counting of PMN lymphocytes and macrophages were carried out in a blinded fashion, at the light of microscope at 100× objective to determine the most common cellular phenotypes activated during the infection. PMN were identified by the shape of their nucleus bi-, tri-, or multi-lobulated, whereas macrophages cells were identified by size and were hemosiderin-laden macrophages.

### 4.6. Immunohistochemistry (IC) and Immunofluorescence (IF)

Four-micron thick lung sections were, deparaffinized and incubated with 10x DIVA Decloaker (Biocare Medical; CA, USA) at a 1:10 dilution. For IC, the slides were washed with PBS (3 × 5 min) peroxidase inhibition was performed with 10% hydrogen peroxide for 30 min, the slides washed with PBS (3 × 5 min) and blocked with PBS 3% BSA for 1 h at room temperature. The slides were incubated with purified primary rabbit anti-Elastin at a 1:200 dilution (Abcam; MA, USA) or goat anti-MMP9 at a 1:100 dilution (Santa Cruz; CA, USA) at 4 °C overnight. Next, the slides were washed with PBS (3 × 5 min) and secondary mouse anti-rabbit at a 1:1500 dilution (Biolegend; CA, USA) or mouse anti-goat at a 1:1500 dilution (Biolegend; CA, USA) were added and incubated for 1 h at room temperature. The slides were washed and incubated for 5 min with 50 µL of diaminobenzidine (DAB) chromogen kit (Biocare Medical; CA, USA) according to the manufacturer’s instructions. Finally, counterstaining with Harris hematoxylin was performed. Photographs were obtained with an optic microscope (Axio Vert. A1, Carl Zeiss) using a 40× objective and analyzed with the program ImageJ. For IF, the slides were washed with PBS (3 × 5 min) and membrane permeabilization performed with PBS containing 2% Triton (Reasol; Mexico City, Mexico). Sections were washed with PBS (3 × 5 min) and blocked with PBS containing 3% BSA for 1 hour at room temperature. Different tissue sections were incubated with purified primary antibodies labeled with FITC fluorochrome rabbit anti-iNOS at a 1:100 dilution (Cell Signaling; MA, USA) or rabbit anti-Ym1 at a 1:100 dilution (Stem Cell, CA; Canada) overnight at 4 °C. Next, the slides were washed, with distilled water 3 times and mounted with one drop of Fluoroshield mounting medium with DAPI (Abcam; MA, USA) per tissue section. Immunofluorescence was analyzed using an Axio Vert A1 microscope (Zeiss).

### 4.7. ELISA

Peripheral blood was collected from the lateral tail vein and centrifuged at 2500 rpm for 10 min, and serum was collected and tested for *T. canis*-specific IgG1 and IgG2a in antigen-coated plates (1 µg/mL), excreted-secreted antigens of *T. canis* were used to sensitized plated were obtained according to previous protocols [68]. After overnight incubation at 4 °C, the plates were washed with PBS supplemented with 0.05% Tween 20 (Sigma; St. Louise, USA) and blocked with PBS supplemented with 1% BSA (Biowest). Serial dilutions of serum samples starting at 1:100 were added to the plates. Bound antibodies were detected following incubation with HRP-conjugated rat anti-mouse IgG1 or IgG2a (Zymed; San Francisco, USA) and read on a microplate reader at 405 nm (Multiskan Ascent, Thermo Labsystems). The results are expressed as the maximal sera dilution at which optical density (OD) was detected. Serum levels of IFN-γ, TNF-α, IL-4, IL-13, and IL-10 were measured by ELISA (Peprotech; Mexico City, Mexico) according to the manufacturer’s instructions.

### 4.8. RT-PCR Analysis

Lungs were placed in 1.5 mL Eppendorf tubes containing TRIzol reagent (Invitrogen; CA, USA) and 0.5 mm diameter zirconium oxide beads (Next Advance) for tissue digestion in a bullet blender (Next Advance). RNA was extracted by the chloroform technique; once quantified, 1 µg of the product was reverse transcribed using the Superscript II First Strand Synthesis Kit (Invitrogen; CA, USA). The primers used to amplify genes and the melting temperatures are described in Table S1. Amplified products were mixed with loading buffer containing SYBR green and observed in a 1.5% agarose gel (IBI Scientific molecular biology certified) with a DocTM-EZ Gel Documentation System. Images were analyzed using ImageJ, and the expression values were normalized to glyceraldehyde 3-phosphate dehydrogenase (GAPDH) as a control (Table S1).

### 4.9. Flow Cytometry

Lungs obtained at 7 dpi were injected with saline solution to obtain cells. Red blood cells were lysed, and live cells were counted using trypan blue exclusion (Countess II FL ThermoFisher). The cells were stained for expression of surface markers using the following antibodies: anti-F4/80-Pacific blue or APC, anti-CD86-PE/Cy7, anti-CD206 (MMR)-PerCP or FITC and anti-IL4Rα-PE (all from Biolegend; CA, USA). The samples were incubated for 30 min at 4 °C in FACS sheet buffer (BD^®^). The samples were acquired using either a FACSAria Fusion (BD^®^) or Attune NxT (ThermoFisher) and analyzed using FlowJo software (version 10.0.7).

### 4.10. Macrophage Depletion with Clodronate Liposomes

We administered clodronate-loaded liposomes intratracheally according to the manufacturer’s instructions (Formumax; CA, USA) to deplete macrophages in the lung tissue. Two days after liposome treatment, mice were infected as described. At day four after the first treatment, a second dose of 100 µL of clodronate-loaded or PBS-loaded liposomes was administered to the corresponding group. At 4 dpi, the animals were euthanized.

### 4.11. Statistical Analysis

Differences between groups were determined by either two-tailed unpaired Student’s t-test or two-way analysis of variance (ANOVA), with multiple Holm–Sidak test, and reported as the means ± SEM. All statistical analyses were determined using GraphPad Prism v 6.0, with a “*p*” value or less than 0.05 considered significant (* *p* < 0.05). All experiments were carried out independently at least twice.

## Supplementary Materials

The following are available online at https://www.mdpi.com/2076-0817/8/4/280/s1, Figure S1: Collagen deposition, Figure S2: M1 and M2 markers after macrophages depletion, Figure S3: Numbers of cells in lung tissue, Table S1: Genes and their respective sequences used to determine macrophage and immune response activation.
