# Supplementary material for: A Dual Role for Macrophages in Modulating Lung Tissue Damage/Repair during L2 Toxocara canis Infection
Source: Pathogens. 2019 Dec 2;8(4):280. doi: 10.3390/pathogens8040280 (PMC6963574; doi:10.3390/pathogens8040280)
Supplement: Supplementary file 1 [file pathogens-08-00280-s001.pdf]

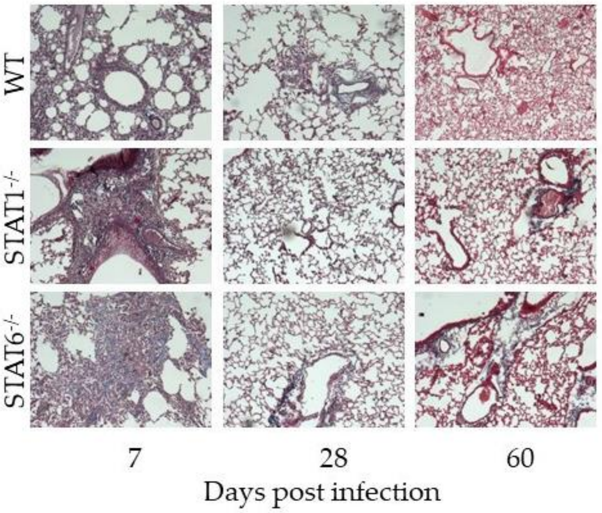

Figure S1. Collagen deposition. Paraffin sections of 4μm thick from lung tissue, were Masson´s Trichromic stained and observed in microscopy light. Images were photographed with the 20× objective at 7, 28 and 60 dpi in WT, STAT1$^{-/-}$, and STAT6$^{-/-}$ mice and collagen deposition was evaluated.

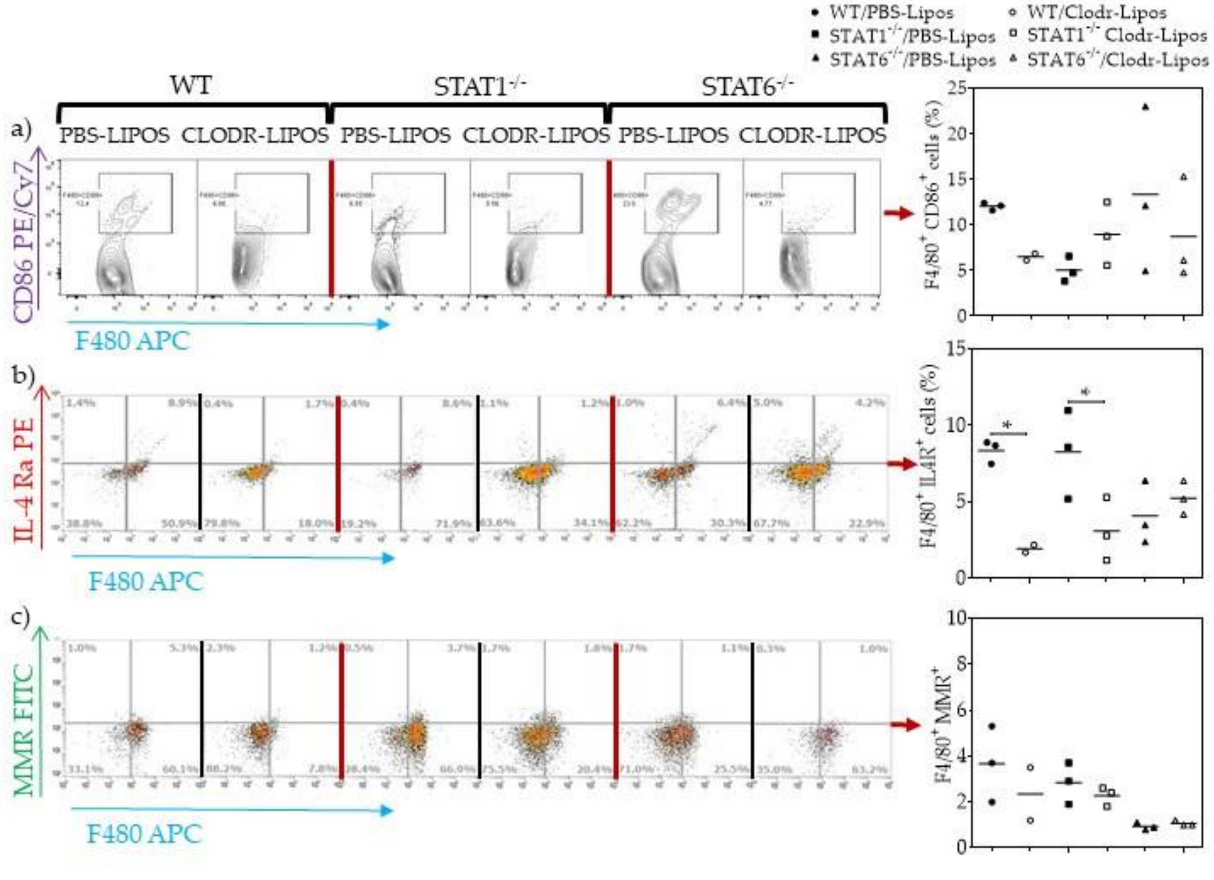

Figure S2. M1 and M2 markers after macrophages depletionn. WT, STAT1$^{-/-}$ and STAT6$^{-/-}$ mice were treated either with PBS-liposomes or clodronate-liposomes intratracheally to deplete macrophages, before and after being infected with 500 L2 T. canis larvae. The animals were euthanized at 4 dpi, lungs cells were collected, and flow cytometry was used to determine M1 or M2 activation. Representative dot plots and their respective percentage of F4/80^{+}CD86^{+} **(a)** F4/80^{+}IL-4Rα^{+} **(b)** and F4/80^{+}MMR^{+} **(c)** double-positive cells are shown. Each dot plot represents an individual mouse. One-way ANOVA and Tukey's multiple comparison test. *P<0.05 comparing WT versus STAT1$^{-/-}$ and STAT6$^{-/-}$ mice.

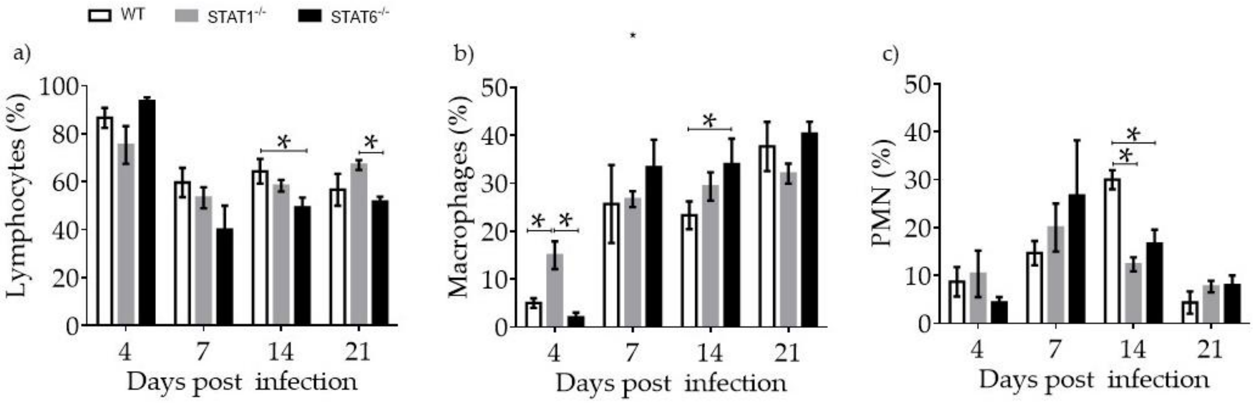

Figure S3. Numbers of cells in lung tissue. From inflammatory infiltrate in H&E stained sections the number of lymphocytes **(a),** macrophages (b) and PMN (c) was quantified in microscopy light (100x objective) images in WT (white bars), STAT1$^{-/-}$ (gray bars) and STAT6$^{-/-}$ (black bars) mice. Data are shown from two independent experiments as mean ± SEM (n = 6 per group). Two-way ANOVA with Tukey multi-comparison test. **P* < *0.05* comparing WT versus STAT1$^{-/-}$ and STAT6$^{-/-}$, and STAT1$^{-/-}$ versus STAT6$^{-/-}$ mice, at the same time point of infection.

Table 1. Genes and their respective sequences used to determine macrophage activation and immune response

Gene	Temperature (°C)	Cycles	Sequence
			Control
GAPDH	54	35	F- CTC ATG ACC ACA GTC CAT GC<br>R-CAC ATT GGG GGT AGG AAC AC
			Th1 Cytokines
IFNγ	57	35	F-AGC GGC TGA CTG AAC TCA GAT TGT AG<br>R-GTC ACA GTT TTC AGC TGT ATA GGG
TNFα	59	35	F-GGC AGG TCT ACT TTG GAG TCA TTGC<br>R-ACA TTC GAG GCT CCA GTG AAT TCG
			Th2 cytokines
IL-4	58	35	F-CGAAGA ACA CCA CAG AGA GTG AGCT<br>R-GAC TCA TTC ATG GTG CAG CCT ATCG
IL-10	56	35	F-ACC TGG TAG AAG TGA TGC CCC AGG CA<br>R-CTA TGC AGT TGA AGA TGT CAA A
			M2 Markers
Arg1	54	35	F-CAG AAG AAT GGA AGA GTC AG<br>R-CAG ATA TGC AGG GAG TCA CC

FIZZ1	62	35	F-GGTCCCAGTGCATATGGATGAGACCATAGA<br>R-CACCTCTTCACTCGAGGGACAGTTGGCAGC
Ym1	56	35	F-TCACAGGTCTGGCAATTCTTCTG<br>R-TTTGTCCTTAGGAGGGCTTCCTC
TGF-β	60	35	F-GCCCTTCCTGCTCCTCAT<br>R-TTGGCATGGTAGCCCTTG
M1 Marker			
iNOS	65	35	F-CTGGAG GAG CTC CTG CCT CATG<br>R-GCA GCA TCC CCT CTG ATG GTG

F, forward; R, reverse primer.
